# Genetic variants in epoxyeicosatrienoic acid processing and degradation pathways are associated with gestational diabetes mellitus

**DOI:** 10.1186/s12937-023-00862-9

**Published:** 2023-06-28

**Authors:** Siyu Lai, Dandan Yan, Jie Xu, Xiangtian Yu, Jingyi Guo, Xiangnan Fang, Mengyang Tang, Rong Zhang, Hong Zhang, Weiping Jia, Mingjuan Luo, Cheng Hu

**Affiliations:** 1 The Third School of Clinical Medicine, Southern Medical University, Guangzhou, China; 2 Department of Endocrinology and Metabolism, Southern Medical University Affiliated Fengxian Hospital, Shanghai, China; 3 Shanghai Diabetes Institute, Shanghai Key Laboratory of Diabetes Mellitus, Shanghai Clinical Center for Diabetes, Shanghai Sixth People’s Hospital Affiliated to Shanghai Jiao Tong University School of Medicine, Shanghai, China; 4 Clinical Research Center, Shanghai Sixth People’s Hospital Affiliated to Shanghai Jiao Tong University School of Medicine, Shanghai, China; 5 Department of Endocrinology, First Affiliated Hospital of Gannan Medical University, Ganzhou, China; 6 Department of Endocrinology and Metabolism, The University of Hong Kong-Shenzhen Hospital, Shenzhen, China

**Keywords:** Gestational diabetes mellitus, Epoxyeicosatrienoic acid, Genetics

## Abstract

**Aim:**

To explore the genetic effects of *CYP2C8*, *CYP2C9*, *CYP2J2*, and *EPHX2*, the key genes involved in epoxyeicosatrienoic acid processing and degradation pathways in gestational diabetes mellitus (GDM) and metabolic traits in Chinese pregnant women.

**Methods:**

A total of 2548 unrelated pregnant women were included, of which 938 had GDM and 1610 were considered as controls. Common variants were genotyped using the Infinium Asian Screening Array. Association studies of single nucleotide polymorphisms (SNPs) with GDM and related traits were performed using logistic regression and multivariable linear regression analyses. A genetic risk score (GRS) model based on 12 independent target SNPs associated with GDM was constructed. Logistic regression was used to estimate odds ratios and 95% confidence intervals, adjusting for potential confounders including age, pre-pregnancy body mass index, history of polycystic ovarian syndrome, history of GDM, and family history of diabetes, with GRS entered both as a continuous variable and categorized groups. The relationship between GRS and quantitative traits was also evaluated.

**Results:**

The 12 SNPs in *CYP2C8*, *CYP2C9*, *CYP2J2*, and *EPHX2* were significantly associated with GDM after adjusting for covariates (all *P* < 0.05). The GRS generated from these SNPs significantly correlated with GDM. Furthermore, a significant interaction between *CYP2J2* and *CYP2C8* in GDM (*P*_Interaction_ = 0.014, OR_Interaction_= 0.61, 95%CI 0.41–0.90) was observed.

**Conclusion:**

We found significant associations between GDM susceptibility and 12 SNPs of the four genes involved in epoxyeicosatrienoic acid processing and degradation pathways in a Chinese population. Subjects with a higher GRS showed higher GDM susceptibility with higher fasting plasma glucose and area under the curve of glucose and poorer β-cell function.

**Supplementary Information:**

The online version contains supplementary material available at 10.1186/s12937-023-00862-9.

## Introduction

Gestational diabetes mellitus (GDM) is defined as diabetes first diagnosed in the second or third trimester of pregnancy that was not clearly overt prior to gestation [1]. The prevalence of GDM varies in different populations and based on diagnostic criteria being used. The latest report from the International Diabetes Federation (IDF) Atlas indicated that the global standardised prevalence of GDM was 14% after adjusting for age [2]. It is estimated that 21.1 million (16.7%) live births to women in 2021 were affected by any type of hyperglycaemia in pregnancy (HIP), of which 80.3% were due to GDM [3]. Against the backdrop of the escalating obesity epidemic and advanced maternal age, the prevalence of GDM has rapidly increased and continues to surge. The importance of detecting GDM is exemplified by the fact that the hyperglycaemia status during pregnancy not only increases the maternal risk of subsequent progression to type 2 diabetes (T2D) by approximately 10 folds [4], compared with healthy controls but also predisposes the offspring to poor metabolic conditions in later life [5, 6]. This contributes to a vicious intergenerational cycle of diabetes and obesity that can impact the global health.

The well-documented risk factors contributing to GDM include maternal features (for example, advanced maternal age, weight, and high parity), previous GDM, and family history of diabetes [7]. Ethnicity has also been shown to be an independent determinant of GDM [8]. Additionally, polycystic ovarian syndrome (PCOS) has been frequently reported to be associated with an elevated risk of GDM [9–11]. Several pathogenic processes are involved in the development of GDM such as pancreatic islet β-cell dysfunction and chronic insulin resistance during pregnancy [12]. In addition, an imbalance between pro-inflammatory and anti-inflammatory processes leads to the progression of GDM. Various inflammatory factors, such as interleukin (IL)-1β, IL-6, IL-8, and tumour necrosis factor alpha, have been confirmed to have an independent positive correlation with GDM [13–16]. Accumulating evidence indicate that GDM is associated with strong genetic predisposition. Over the past few decades, gene loci responsible for insulin secretion and resistance and lipid and glucose metabolism have been found to be associated with GDM [17]. However, its genetics is complex and not fully defined.

Epoxyeicosatrienoic acids (EETs), metabolites of arachidonic acid produced by cytochrome P450 enzymes (CYP450), exhibit multiple biological activities, including anti-inflammatory, vasodilatory, and electrophysiological effects [18–21]. Several in vitro and animal studies have suggested that CYP450-derived EETs exert protective effects on insulin sensitivity and glucose metabolism, which are critical processes in GDM development [22–24]. In humans, the predominant epoxygenases involved in EET formation are CYP2J2, CYP2C8, and CYP2C9, which are encoded by the corresponding genes. Highly unstable EETs are hydrolysed to less active dihydroxyeicosatrienoic acids (DHETs) by soluble epoxide hydrolase (sEH) encoded by *EPHX2* [25, 26]. Previous studies have shown that single nucleotide polymorphisms (SNPs) in *CYP2J2*, *CYP2C8*, *CYP2C9*, and *EPHX2* are associated with diabetes and diabetic kidney disease (DKD); however, their genetic effects on GDM remain unclear.

Therefore, to address this knowledge gap, the present study was designed to investigate the association of common genetic variants of *CYP2J2*, *CYP2C8*, *CYP2C9* and *EPHX2* with GDM, with the aim of providing novel genetic basis for GDM susceptibility.

## Materials and methods

### Ethics statement

The present study was approved by the Institutional Review Board (IRB) of the University of Hong Kong-Shenzhen Hospital ([2017]13) and conducted according to the principles of the Declaration of Helsinki as revised in 2013. Written informed consent was obtained from each participant prior to enrolment.

### Study design and participants

A total of 2548 Chinese women in early pregnancy were recruited between January 2016 and December 2018 at the University of Hong Kong-Shenzhen Hospital. All participants routinely underwent a standard 75-g oral glucose tolerance test (OGTT) at 24–28 weeks of gestation after an overnight fast of at least eight hours [28]. According to the criteria recommended by the International Association of Diabetes and Pregnancy Study Groups (IADPSG) [27], GDM was diagnosed if any of the following threshold values were equalled or exceeded: fasting plasma glucose: 5.1 mmol/L (92 mg/dL), one-hour plasma glucose (1 h-PG): 10.0 mmol/L (180 mg/dL), or two-hour plasma glucose (2 h-PG): 8.5 mmol/L (153 mg/dL). Participants with diabetes antedating pregnancy were excluded. 

### Clinical measurements

Information on demographics, family, and medical history of PCOS and GDM was collected using a standard questionnaire. Weight and standing height were measured with light clothes and no shoes, according to standard protocols by trained investigators [29, 30]. Pre-pregnancy weight was self-reported, and pre-pregnancy body mass index (BMI; kg/m^2^) was calculated as pre-pregnancy weight (kg) divided by the square of height (m). Blood pressure measurements were performed in both arms using a mercury sphygmomanometer after a rest period of at least 5 min, and the arm with the higher reading was tested twice at 3-min intervals to calculate the mean value [31]. Blood samples collected in the fasting status were used to measure the levels of fasting plasma glucose (FPG), fasting insulin (FINS), glycated haemoglobin A1c (HbA1c), total cholesterol, triglyceride, high-density lipoprotein cholesterol (HDL-C), and low-density lipoprotein cholesterol (LDL-C); details of these biochemical measurements have been described previously by Lu W et al. [32, 33]. Homeostasis model assessment of β-cell function (HOMA-β) and insulin resistance (HOMA-IR) were used to evaluate basal insulin secretion and insulin resistance, respectively, which were calculated using insulin and glucose concentrations as follows: HOMA-β = 20 × FINS (mU/L)/[FPG (mmol/L) − 3.5] and HOMA-IR = FINS (mU/L)/(22.5e^− lnFPG (mmol/L)^) [34, 35]. The area under curve of glucose (G_AUC_) from the 75-g OGTT was calculated as 1/2 × [FPG (mmol/L) + 1 h-PG (mmol/L)] × 1 h + 1/2 × [1 h-PG (mmol/L) + 2 h-PG (mmol/L)] × 1 h [36]. In addition, PCOS was diagnosed according to the revised 2003 consensus [37]. The birth of an infant before completion of 37 weeks of gestation was defined as preterm delivery. Macrosomia, preterm delivery, and caesarean delivery referred to current pregnancies.

### SNP genotyping and quality control analysis

**Fig. 1:**
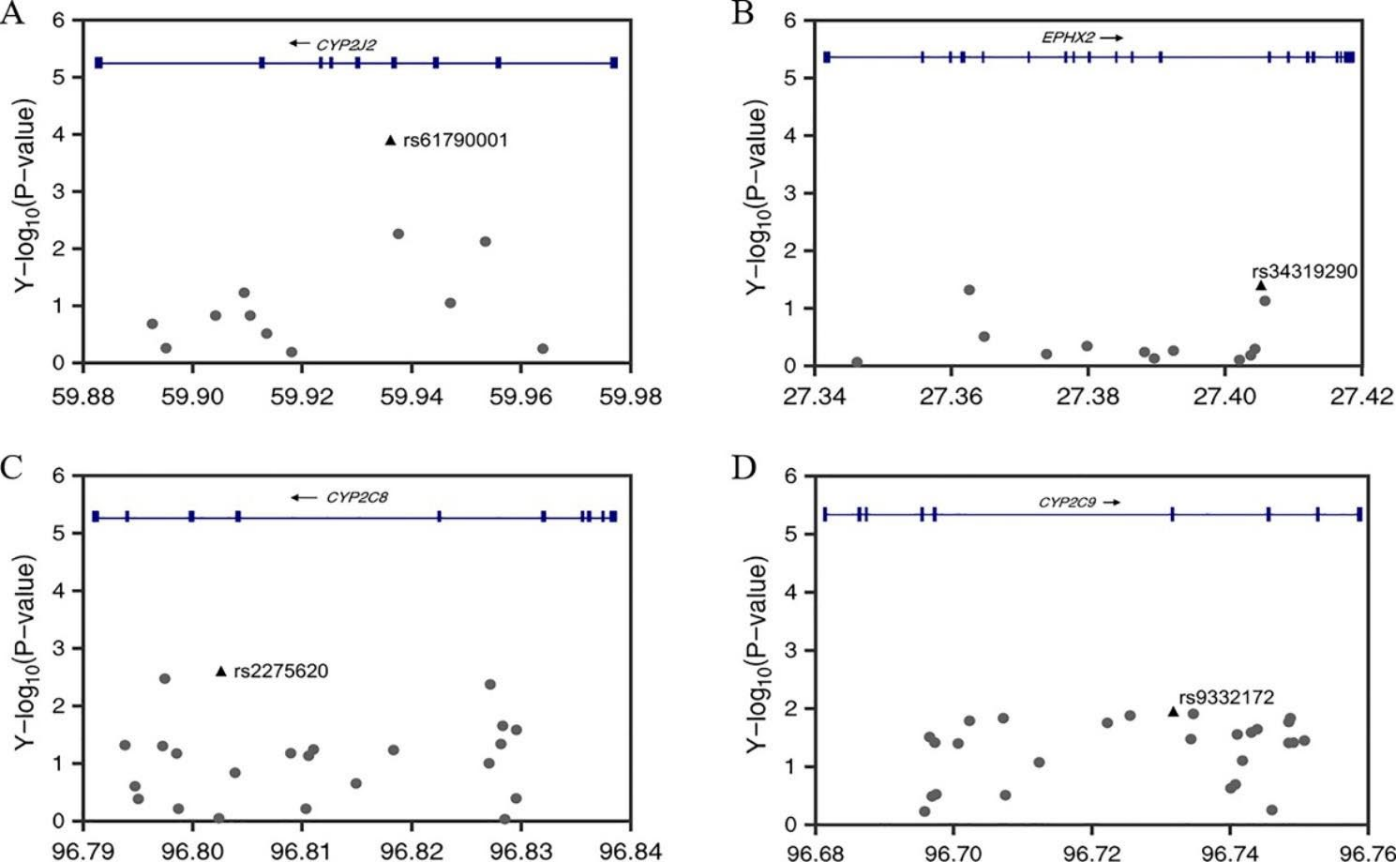
Regional association plot of SNPs in *CYP2J2* (**A**),*EPHX2* (**B**), *CYP2C8* (**C**), and *CYP2C9* (**D**) with GDM. The most signifcant SNP of each gene is highlighted in black triangle. The -log_10_*P*-value for the associations are given at the y-axis and the chromosomal positions (Genome Reference Consortium Human Build 37, GRCh37) of the SNPs are plotted

DNA was extracted from the peripheral blood using DNA Extraction Kit (Qiagen, Duesseldorf, Germany) according to the manufacturer’s instructions. The concentration of DNA in each sample was measured using NanoDrop2000 Spectrophotometer (Thermo Fisher Scientific, Waltham, MA). A 260/280 ratio of ~ 1.8 was generally accepted as “pure” for DNA. Genotyping was performed using Infinium Asian Screening Array-24 v1.0 BeadChip (Illumina, Inc., San Diego, CA, United States). Four critical genes related to EET processing and degradation pathways were selected. Genetic variants located within 3 kb upstream and 3 kb downstream of each gene region were extracted for further analysis [Fig. 1]. All single nucleotide polymorphisms (SNPs) were filtered based on the Hardy-Weinberg equilibrium in controls (HWE; *P* < 0.01), minor allele frequency (MAF > 0.001), and success rate (> 0.97) using the PLINK version 1.9 (https://www.cog-genomics.org/plink/1.9/) [38]. Pairwise linkage disequilibrium (LD) analysis was performed to obtain independent target SNPs using the Haploview version 4.2 (https://www.broadinstitute.org/haploview/downloads) [39]. To avoid the inflation of the estimates due to linkage disequilibrium, the threshold of r^2^ < 0.49 was used to select independent SNPs. Quality control procedures for individual samples required a call rate > 95%.

### Statistical analysis

All data were collected on standard forms, checked for completeness, double-entered, compared, and corrected for inconsistencies. The normality of the distribution of continuous variables was tested using the Kolmogorov–Smirnov test. Continuous variables with normal distribution, that is, age, systolic blood pressure, and diastolic blood pressure were presented as the mean ± standard deviation (SD), and variables with non-normal distribution were reported as the median (interquartile range). Frequencies and proportions were used for categorical variables. The differences in mean, median, or frequency between groups were compared using independent samples Student’s *t*-test, Mann–Whitney *U* test, Kruskal–Wallis test, or Pearson χ^2^ test, as appropriate. The HWE in the control group was calculated using chi-square goodness of fit test. Genotype and allele distributions were compared using χ^2^ test. Data with skewed distribution were logarithmically transformed before analysis. Association tests were performed within a linear or logistic regression framework using the PLINK software. An additive genetic model was used to analyse allele dosage in which the genotypes AA, Aa, aa were coded as 0, 1, and 2, respectively (‘A’ represents the common allele and ‘a’ represents the rare allele). Age, pre-pregnancy BMI, history of PCOS, history of GDM and family history of diabetes were included as covariates and adjusted using a multivariable regression model; unadjusted and adjusted odds ratios (OR) and 95% confidence intervals (95% CI) were calculated. Gene-gene interaction (SNP-SNP epistasis) were performed by PLINK with a model based on allele dosage for each SNP, A and B, and fits in the form of ‘Y ~ b0 + b1.A + b2.B + b3.AB + e’. The test for interaction is based on the coefficient b3. All pairwise combinations of SNPs were tested. Multiple testing corrections were made using the Benjamini-Hochberg false discovery rate (FDR) method with a threshold of 0.05 for statistical significance [40].

The number of risk alleles (zero, one, or two) is summed for 12 independent variants, to generate unweighted genetic risk scores (GRS). Moreover, a weighted GRS was generated for each individual by taking the sum of the weighted number of observed risk alleles, each risk allele weighted by SNP-specific per allele effect size (log_e_[OR]) from the single SNP analysis, and dividing by the mean per allele effect size for the SNPs. Association between GRS and GDM susceptibility was evaluated [41]. To better interpret the results, the subjects were stratified into five GRS groups considering the distribution of GRS. We performed logistic regression analyses of the GRS groups against GDM case-control status and GRS group (1,2,3,4,5) was entered as a continuous variable. The association between GRS group and traits were analysed using linear regression. To better reflect the changing trend of metabolic traits across the GRS groups, trait levels were normalised using Z-scores, which were calculated by subtracting the mean from the raw score and then dividing the difference by the standard deviation [42]. Restricted cubic splines (RCS) were implemented to detect potential nonlinear association of GRS with GDM and metabolic traits, and the RCS models were adjusted for covariates. All statistical analyses were performed using the IBM SPSS Statistics for Windows (version 25.0; IBM Corp., Armonk, New York, USA) and R software v.4.1.3. A *P*-value of less than 0.05 (two-sided test of significance) was considered statistically significant.

## Results

### Subject characteristics

The clinical and demographic characteristics of the two groups are shown in Table 1. In total, 2548 participants were included in our study. Among them, 938 were diagnosed with GDM, and 1610 with normal glucose tolerance (NGT) were considered as controls. The mean age was 31.79 year (± 4.08), and the median gestational age at delivery was 39.29 weeks (38.57–40.00). Compared with the control group, pregnant women with GDM were older and more likely to report PCOS history, GDM history and family history of diabetes. Additionally, pre-pregnancy BMI, blood pressure, lipid parameters (total cholesterol, triglyceride, LDL-C, and HDL-C), and glycaemic parameters (FPG, 1 h-PG, 2 h-PG, G_AUC_, HbA1_c_, and HOMA-IR) were significantly higher, and BMI changes during pregnancy and HOMA-β were significantly lower in the GDM group than in the control group (all *P* < 0.001). There were no significant differences in the multiparous status, macrosomia, or primary caesarean delivery between the two groups (all *P* > 0.05).

**Table 1:** Clinical characteristics of the GDM and control groups

Traits	Overall (n = 2548)	Control (n = 1610)	GDM (n = 938)	*P*
Age,yr	31.79 ± 4.08	31.59 ± 4.05	32.14 ± 4.11	***0.001***
Prepregnancy BMI,kg/m2	20.96 (19.30–22.85)	20.81 (19.22–22.66)	21.23 (19.53–23.22)	***< 0.001***
Gestational age at delivery,weeks	39.29 (38.57,40.00)	39.43 (38.71–40.14)	39.14 (38.29–40.00)	***< 0.001***
BMI change,kg/m2	5.02 (3.95–6.09)	5.34 (4.35–6.29)	4.43 (3.33–5.55)	***< 0.001***
Systolic blood pressure, mmHg	109.08 ± 10.28	108.25 ± 9.86	110.50 ± 10.82	***< 0.001***
Diastolic blood pressure, mmHg	66.93 ± 8.20	66.27 ± 7.90	68.06 ± 8.57	***< 0.001***
Multiparous, n (%)	1167 (45.8)	727 (45.2)	440 (46.9)	*0.392*
History of GDM, n (%)	81 (3.2)	11 (0.7)	70 (7.5)	***< 0.001***
History of PCOS, n (%)	26 (1.0)	3 (0.2)	23 (2.5)	***< 0.001***
Family history of diabetes,n (%)	296 (11.6)	72 (4.5)	224 (23.9)	***< 0.001***
FPG, mmol/L	4.47 (4.24–4.75)	4.39 (4.18–4.61)	4.68 (4.38–5.11)	***< 0.001***
1 h-PG, mmol/L	8.28 (7.01–9.63)	7.50 (6.52–8.38)	10.06 (9.12–10.63)	***< 0.001***
2 h-PG, mmol/L	7.09 (6.11–8.44)	6.46 (5.76–7.19)	8.79 (7.98–9.44)	***< 0.001***
G_AUC_, mmol/L·h	14.09 (12.39–16.05)	12.89 (11.73–14.13)	16.54 (15.67–17.55)	***< 0.001***
DCCT-HbA1c,^a^ %	5.2 (5.0–5.4)	5.2 (5.0–5.3)	5.3 (5.1–5.5)	***< 0.001***
IFCC-HbA1c,^b^ mmol/mol	33.3 (31.1–35.5)	33.3 (31.1–34.4)	34.4 (32.2–36.6)	
Fasting insulin, mU/L	8.96 (6.18–12.77)	8.85 (6.04–12.76)	9.10 (6.40–12.80)	*0.126*
HOMA-β	188.98 (129.13–271.40)	208.77 (145.65–297.65)	158.38 (109.50–228.50)	***< 0.001***
HOMA-IR	1.79 (1.19–2.60)	1.71 (1.16–2.51)	1.90 (1.29–2.80)	***< 0.001***
Total cholesterol, mmol/L	5.70 (4.96–6.66)	5.63 (4.87–6.43)	5.79 (5.10–6.50)	***< 0.001***
Triglyceride, mmol/L	2.17 (1.71–2.83)	2.07 (1.64–2.73)	2.32 (1.88–2.94)	***< 0.001***
LDL-C, mmol/L	2.94 (2.42–3.55)	2.80 (2.32–3.35)	3.24 (2.62–3.91)	***< 0.001***
HDL-C, mmol/L	1.88 (1.61–2.16)	1.84 (1.58–2.12)	1.95 (1.66–2.25)	***< 0.001***
Initial cesarean section ^c^, n/total n(%)	405/2045 (19.8)	254/1299 (19.6)	151/746 (20.2)	*0.707*
Preterm delivery ^d^, n(%)	124 (4.9)	62 (3.9)	62 (6.7)	***0.002***
Macrosomia ^e^, n(%)	139 (5.5)	88 (5.5)	51 (5.5)	*0.999*
Insulin therapy, n(%)	97 (3.8)	-	97 (10.3)	***< 0.001***

Data are presented as the mean ± SD, median (interquartile range), or n (%). The Wilcoxon test was used for continuous variables with skewed distribution. The chi-square test was used for categorical variables. *P* values < 0.05 are written in bold letters.

**Abbreviations**: BMI, body mass index; PCOS, polycystic ovary syndrome; FPG, fasting plasma glucose; 1 h-PG, oral glucose tolerance test (OGTT) one-hour plasma glucose; 2 h-PG, OGTT two-hour plasma glucose; G_AUC_, area under the curve of glucose from the 75-g OGTT; HbA1c, haemoglobin A1c; HDL-C, high-density lipoprotein cholesterol; LDL-C, low-density lipoprotein cholesterol; HOMA-β, homeostasis model assessment index of β-cell secretion; HOMA-IR, homeostasis model assessment of insulin resistance.

^a^ Diabetes Control and Complications Trial (DCCT) units of HbA1c values. ^b^ International Federation of Clinical Chemistry (IFCC) units of HbA1c values.^c^ woman without a history of caesarean section. ^d^ birth of an infant before completion of 37 weeks of gestation is defined as preterm delivery. ^e^ Excessive birth weight, usually defined as > 4000.

### Association between SNPs and GDM

We first examined the potential effects of these SNPs on GDM susceptibility in the study population. After the quality control procedure, 31 SNPs were included in the LD analysis [Suppl. Figure 1]. Among these, 12 SNPs were selected for further analysis (r^2^ < 0.49). Table 2 presents the genotype and allele distribution of the 12 independent SNPs and the corresponding odds ratios for GDM. The A allele of rs61790001 in *CYP2J2* was the most significant SNP associated with GDM in the unadjusted model (OR 0.73 [95% CI 0.62–0.86], *P* = 0.0001). Considering the confounders, only 7 SNPs (rs61790001, rs76271683, rs57699806, rs11572177, rs9332092, rs4918758, and rs2860905) retained significance after multiple testing corrections (FDR < 0.05). The missense variant rs57699806 in *EPHX2* was associated with GDM (adjusted OR 1.46 [95% CI 1.10–1.93], FDR = 0.044). Moreover, only rs61790001 in *CYP2J2* presented a decresed risk of GDM (adjusted OR 0.73 [95% CI 0.61–0.86]). *CYP2J2*-rs76271683 and *CYP2C8*-rs11572177 were associated with an increased risk of GDM (adjusted OR 1.27 [95% CI 1.07–1.49], 1.34[95% CI 1.07–1.67]; FDR = 0.028, 0.046, respectively). The minor allele frequency(MAF) of these 12 SNPs in the current study and in other populations is shown in Suppl. Table 1.

**Table 2:** Association between SNPs and GDM

SNP	Chr:bp ^a^	Gene	Allele	MAF	Variant type	Frequency			Genotype count					
						Case/ Control	OR (95% CI)	*P*-allele	FDR	Case	Control	OR (95% CI)	*P*-genotype	FDR
rs61790001	1:59936155	*CYP2J2*	A/G	0.153	intron	0.128/0.168	0.73 (0.62–0.86)	*0.0001*	**0.0094**	22/196/720	45/451/1113	0.73 (0.61–0.86)	*0.0003*	**0.0026**
s144619025	1:59937579	*CYP2J2*	A/G	0.035	intron	0.026/0.040	0.62 (0.45–0.87)	*0.0055*	0.0835	0/48/890	4/122/1483	0.68 (0.48–0.97)	*0.0354*	0.1105
rs76271683	1:59953494	*CYP2J2*	G/A	0.167	intron	0.186/0.157	1.23 (1.06–1.43)	*0.0075*	0.0950	27/294/617	36/432/1142	1.27 (1.07–1.49)	*0.0048*	**0.0280**
rs57699806	8:27362587	*EPHX2*	A/G	0.047	missense	0.054/0.042	1.30 (1.002–1.696)	*0.0479*	0.1211	3/96/838	3/130/1475	1.46 (1.10–1.93)	*0.0087*	**0.0440**
rs34319290	8:27405207	*EPHX2*	A/G	0.098	downstream	0.110/0.092	1.22 (1.01–1.47)	*0.0393*	0.1115	11/183/740	9/275/1311	1.27 (1.04–1.56)	*0.0210*	0.0751
rs11572177	10:96797270	*CYP2C8*	G/A	0.076	intron	0.085/0.070	1.24 (1.00–1.53)	*0.0494*	0.1211	6/148/784	8/210/1392	1.34 (1.07–1.67)	*0.0111*	**0.0462**
rs1934956	10:96828160	*CYP2C8*	A/G	0.455	intron	0.437/0.466	0.89 (0.793–0.998)	*0.0454*	0.1211	172/475/291	351/796/462	0.87 (0.77–0.99)	*0.0324*	0.1045
rs2071426	10:96828323	*CYP2C8*	G/A	0.068	intron	0.079/0.062	1.29 (1.04–1.61)	*0.0220*	0.1075	2/144/792	8/184/1418	1.35 (1.06–1.70)	*0.0139*	0.0533
rs9332092	10:96696529	*CYP2C9*	G/A	0.045	upstream	0.053/0.040	1.34 (1.03–1.75)	*0.0308*	0.1115	0/100/835	0/130/1478	1.54 (1.15–2.06)	*0.0033*	**0.0226**
rs4918758	10:96697252	*CYP2C9*	G/A	0.390	upstream	0.408/0.379	1.13 (1.007–1.270)	*0.0384*	0.1115	149/467/321	222/776/612	1.18 (1.04–1.34)	*0.0102*	**0.0447**
rs2860905	10:96702295	*CYP2C9*	A/G	0.096	intron	0.109/0.088	1.26 (1.044–1.526)	*0.0161*	0.0954	10/184/744	14/255/1336	1.36 (1.11–1.66)	*0.0032*	**0.0226**
rs9332146	10:96722244	*CYP2C9*	A/G	0.031	intron	0.023/0.035	0.65 (0.460–0.931)	*0.0176*	0.0954	0/44/893	2/110/1496	0.65 (0.44–0.95)	*0.0256*	0.0883

**Abbreviations**: Chr, chromosome; SNP, single-nucleotide polymorphism; Allele, minor/major allele; MAF, minor allele frequency; OR, odds ratios; 95% CI, 95% confidence interval; FDR, false discovery rate.

**Note**: The *P*-allele was derived from the comparison of the allele frequency between the GDM and control groups. *P*-genotype refers to the comparison of genotype distribution between the case group and control group, adjusted for maternal age, pre-pregnancy BMI, history of PCOS, history of GDM and family history of diabetes using logistic regression analysis in the additive genetic model; OR with 95% CI shows the association between the effect allele and GDM. FDR values < 0.05 are written in bold letters.

a Positions are based on the Human Genome version 19 (hg19), build 3.

### Association between SNPs and metabolic traits

**Fig. 2:**
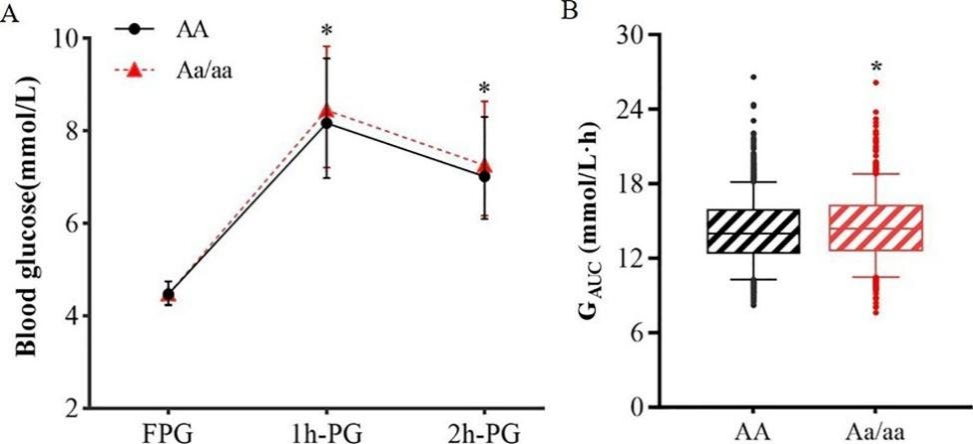
Association between *CYP2J2*-rs76271683 and metabolic traits. Median values and 95% CI of blood glucose (**A**) and box plot of area under the curve of glucose (G_AUC_) (**B**) indicate that the glucose level was significantly higher in the minor allele carrier group. A: major allele; a: minor allele. Within each box, horizontal lines denote median values; boxes extend from the 25th to the 75th percentile of each group’s distribution of values; whiskers above and below the box indicate the 5th and 95th percentiles. Points above and below the whiskers indicate outliers *P < 0.05 vs. AA group after adjusted for maternal age, pre-pregnancy BMI, history of PCOS, history of GDM and family history of diabetes

We subsequently analysed the association between SNPs and metabolic traits. As shown in Fig. 2, *CYP2J2*-rs76271683 was significantly associated with glucose indicators, including 1 h-PG (β = 0.011, SE = 0.003, FDR = 0.0125), 2 h-PG (β = 0.011, SE = 0.003, FDR = 0.0145), and G_AUC_ (β = 0.010, SE = 0.003, FDR = 0.0047) [Suppl. Table 2]. Moreover, *EPHX2*-rs57699806 and *CYP2C8*-rs11572177 were associated with higher level of 1 h-PG and G_AUC_ after multiple testing corrections (all FDR < 0.05), while *CYP2C9-*rs9332146 was in negative association with 1 h-PG and G_AUC_ [Suppl. Figure 2 A-B]. Both rs9332092 and rs2860905 in *CYP2C9* were associated with lower level of HOMA-β (β = -0.048, -0.034; SE = 0.018, 0.012, respectively) [Suppl. Table 3; Suppl. Figure 2D]. In addition, the A allele of *CYP2J2*-rs144619025 was associated with lower LDL-C level (β = -0.033, SE = 0.010, FDR = 0.0164) [Suppl. Table 4; Suppl. Figure 2C]. However, no association was observed between SNPs and fasting plasma glucose, HbA1c, fasting insulin, HOMA-IR, total cholesterol, triglyceride, or HDL-C levels.

### GRS of risk variants associated with GDM and metabolic traits

**Fig. 3:**
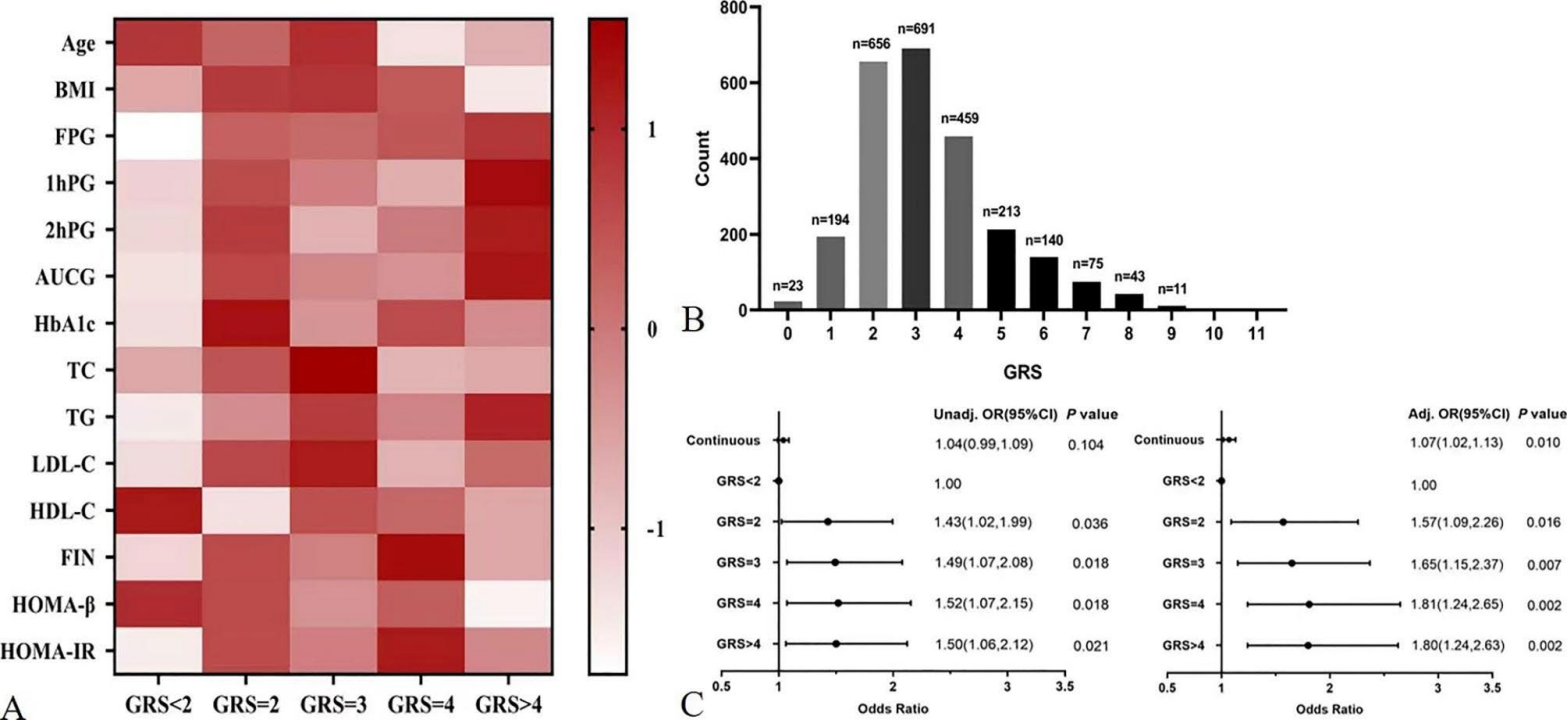
Association of genetic risk score (GRS) based on 12 SNPs with GDM and metabolic traits. Heatmap of metabolic traits z-scores computed for different GRS groups (**A**). Frequency distribution across the GRS groups (**B**). The histogram is plotted on the x-axis representing each GRS category as the sum of the number of risk alleles across the 12 loci, and the y-axis plots the number of individuals in each GRS category. Forest plot of the association between GRS and GDM (**C**). Adjusted OR and P-value refer to adjustment for maternal age, pre-pregnancy BMI, history of PCOS, history of GDM, and family history of diabetes

To evaluate population with higher susceptibility to GDM and determine whether it affected the assessment of GDM and metabolic traits in our study, genetic risk score (GRS) was generated based on the 12 independent SNPs. GRS (continuous) slightly increase the susceptibility to GDM (adjusted OR 1.07 [95% CI 1.02–1.13]). Then, to better interpret the results, the subjects were stratified into five risk groups considering the distribution of GRS [Fig. 3B]. Compared to GRS < 2 group, susceptibility to GDM increased in both unadjusted and adjusted models with the increase in the GRS[Figure 3 C]. The subjects harbouring GRS of 2, 3, 4 and > 4 all presented an increased risk of GDM (adjusted OR 1.57 [95% CI 1.09–2.26], 1.65 [95% CI 1.15–2.37], 1.81 [95% CI 1.24–2.65], and 1.80 [95% CI 1.24–2.63], respectively, all *P* < 0.05). Multivariable linear regression indicated that the median FPG and G_AUC_ increased while the median HOMA-β decreased in higher GRS groups after adjusted for confounders (all *P* < 0.05). The heatmap shows the relative change trend of metabolic traits with the GRS [Fig. 3A] and the characteristics of the GRS groups are presented in Table 3. Overall, individuals with a higher GRS presented a higher susceptibility to GDM, higher FPG and G_AUC_ and impaired insulin secretion than those with a lower GRS.

**Table 3:** Association of GRS based on 12 SNPs with metabolic traits

Traits	GRS < 2 (n = 217)	GRS = 2 (n = 656)	GRS = 3 (n = 691)	GRS = 4 (n = 459)	GRS > 4 (n = 486)	β	SE	*P*
Age, yr	31.94 ± 3.86	31.83 ± 4.14	31.97 ± 4.11	31.52 ± 3.89	31.65 ± 4.21	-	-	*-*
Pre-pregnancy BMI, kg/m2	20.81 (19.53–22.77)	21.21 (19.40–22.86)	21.03 (19.47–22.89)	20.81 (19.23–22.86)	20.77 (19.03–22.66)	-	-	*-*
FPG, mmol/L	4.41 (4.20–4.66)	4.47 (4.25–4.75)	4.47 (4.23–4.76)	4.49 (4.22–4.76)	4.48 (4.25–4.76)	0.002	0.001	***0.002***
1 h-PG, mmol/L	8.30 (7.00–9.46)	8.31 (7.07–9.64)	8.29 (7.10–9.60)	8.16 (6.90–9.68)	8.32 (7.02–9.68)	0.002	0.001	*0.109*
2 h-PG, mmol/L	6.85 (6.01–8.12)	7.11 (6.21–8.51)	7.09 (6.07–8.40)	7.06 (6.10–8.54)	7.23 (6.11–8.49)	0.002	0.001	*0.118*
G_AUC_, mmol/L·h	13.87 (12.29–15.78)	14.16 (12.49–16.06)	14.15 (12.39–16.08)	13.99 (12.31–16.13)	14.12 (12.38–16.17)	0.002	0.001	***0.044***
DCCT-HbA1c, %	5.20 (5.00–5.40)	5.20 (5.00–5.40)	5.20 (5.00–5.38)	5.20 (5.00–5.40)	5.20 (5.00–5.40)	1.92E-4	0.002	*0.662*
Total cholesterol, mmol/L	5.69 (4.98–6.52)	5.72 (4.90–6.45)	5.70 (4.97–6.52)	5.65 (5.00–6.40)	5.70 (4.90–6.40)	1.51E-4	0.003	*0.932*
Triglyceride, mmol/L	2.10 (1.69–2.75)	2.20 (1.74–2.79)	2.16 (1.71–2.85)	2.14 (1.68–2.82)	2.18 (1.71–2.90)	0.004	0.003	*0.172*
LDL-C, mmol/L	2.92 (2.41–3.48)	2.95 (2.44–3.57)	3.00 (2.44–3.62)	2.89 (2.36–3.47)	2.94 (2.45–3.55)	-0.001	0.008	*0.787*
HDL-C, mmol/L	1.91 (1.71–2.18)	1.85 (1.59–2.16)	1.89 (1.61–2.15)	1.90 (1.57–2.20)	1.90 (1.59–2.16)	0.001	0.002	*0.552*
Fasting insulin, mU/L	8.46 (5.98–12.05)	9.52 (6.26–13.37)	8.96 (6.31–12.36)	9.12 (6.26–13.14)	8.53 (5.79–12.54)	-0.002	0.004	*0.620*
HOMA-IR	1.67 (1.15–2.40)	1.90 (1.20–2.71)	1.78 (1.23–2.53)	1.84 (1.19–2.73)	1.71 (1.15–2.58)	6.58E-5	0.004	*0.987*
HOMA-β	193.42 (127.05–275.63)	197.38 (130.54–281.43)	186.62 (128.72–272.83)	195.48 (133.02–275.91)	174.60 (125.05–256.99)	-0.010	0.004	***0.017***

Data are presented as the mean ± SD or median (interquartile range)

The association between GRS groups (1,2,3,4,5) and traits were analysed using linear regression adjusted for maternal age, pre-pregnancy BMI, history of PCOS, history of GDM, and family history of diabetes. *P* values < 0.05 are written in bold letters

### Gene-gene interaction

**Fig. 4:**
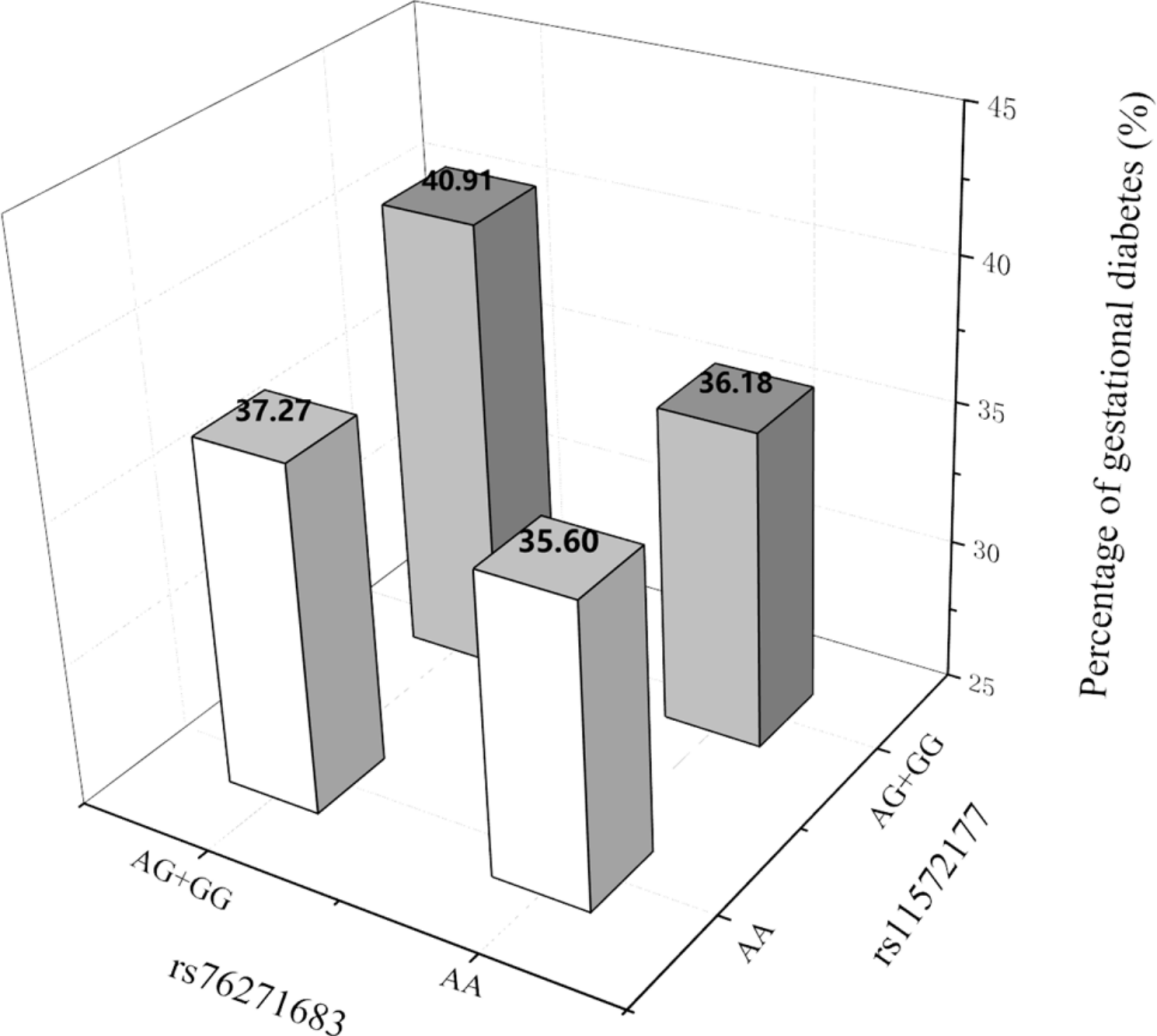
Epistatic analysis between rs76271683 in *CYP2J2* and rs11572177 in *CYP2C8*. The percentage of patients with GDM based on the genotypes is shown

Gene-gene interaction (epistasis) analyses were carried out to avoid overlooking the heritability of GDM due to undiscovered interactions between them. After all pairwise combinations of the 12 SNPs were tested, only *CYP2J2*-rs76271683 and *CYP2C8-*rs11572177 exhibited significant epistatic effects on GDM susceptibility in all subjects (*P*_Interaction_ = 0.014, OR_Interaction_ = 0.61, 95%CI 0.41–0.90) [Suppl.Table 5]. The proportion of patients with GDM increased in subgroups with a greater number of risk alleles [Fig. 4].

## Discussion

Several genome-wide association studies (GWAS) and candidate gene association studies have been performed to examine the association between genetic variants and the risk of GDM, which can be utilised to identify individuals at high risk for GDM or in the early stage of the disease. Notwithstanding, these studies have largely focused on common variants known to be associated with T2D and glycaemic traits outside of pregnancy based on the hypothesis that a shared genetic architecture exists between GDM and T2D [32, 43]. For example, *TCF7L2*, *GCK*, *KCNJ11*, *CDKAL1*, *IGF2BP2*, and *MTNR1B* are thought to modulate pancreatic islet β-cell function, all of which were associated with GDM (OR 1.15–1.46) [44, 45]. EETs are epoxygenase derivatives of arachidonic acid and closely related to pancreatic β-cell function, insulin resistance, glucose homeostasis, and other pathophysiological processes of glucose metabolism. Preclinical studies have consistently shown a protective role of EETs in the aetiology and progression of various metabolic diseases, such as diabetes and its complications [46]. Back in 1983, EETs were shown to stimulate insulin secretion in isolated rat islets and were relatively regioselective for EET formation [47], involving three critical genes (*CYP2C8*, *CYP2C9*, and *CYP2J2*). In addition to production of EETs and anti-inflammatory process, these genes are also involved in both insulin sensitivity in peripheral tissues and the capacity of the islets to respond to insulin resistance [48, 49]. CYP-derived EETs induce insulin secretion and protect pancreatic islet cells from apoptosis [50, 51]. Previous study indicated that *CYP2J3* overexpression improved insulin resistance in rats treated with fructose and in db/db diabetic mice, improving insulin resistance by activating insulin receptor signaling and adiponectin-mediated AMPK signaling pathways [51, 52]. *CYP2J3* gene delivery markedly reversed insulin resistance via upregulated AMPK signaling, which was associated with decreased ER stress response in adipose tissue [51]. The *CYP2C* gene family locus is highly polymorphic. Previous studies have indicated that *CYP2J2 G-50T* polymorphism *(rs890293)* was significantly associated with younger onset (less than 40 years old) T2D in a Chinese population. *CYP2C8-*rs10509681 was associated with an increased risk of DKD [53]. Our results demonstrated that both *CYP2J2*-rs76271683 and *CYP2C8*-rs11572177 were associated with an increased risk of GDM. In addition, the G allele of rs76271683 was associated with glucose metabolism indicators, including 1 h-PG, 2 h-PG, and G_AUC_. *EPHX2* encodes the enzyme (sEH) responsible for the hydrolysis of EETs. *EPHX2* rs751141 (Arg287Gln) polymorphism has been reported to be associated with insulin resistance in patients with T2D in Japanese population [54]. In our study, we found that the missense variant rs57699806 in *EPHX2* was not only associated with an increased risk of GDM, but also with a higher level of 1 h-PG and G_AUC_.

GDM appears to influence the transfer of PUFAs from mothers to fetuses, which may affect the development of the fetal brain and impair the cognitive ability of the infant [55]. Ortega-Senovilla et al. proposed that there is a higher requirement for maternal fatty acids (both AA and DHA) in the fetuses of women with GDM than in those without GDM [56]. We found that individuals with certain variants in genes involved in epoxyeicosatrienoic acid (EET) processing and degradation pathways showed higher susceptibility to GDM. Considering variants may affect activity or yield of cytochrome P450 enzymes, and thus the substrate arachidonic acid content, individuals with certain variants may need to supplement more arachidonic acid, especially for pregnant women.

The discovery of multiple loci associated with GDM has demanded investigation of its clinical implications. The most common disease-associated genetic variants have a small effect size and are likely to explain only a limited fraction of heritability [43]. Thus, we attempted to aggregate the information of variants by constructing genetic risk scores. Weighted GRS is often based on the effect size reported in prior GWAS summary statistics. Therefore, a simple GRS was first used because for the risk alleles included there was inadequate information in the literature to assign a weight. An unweighted GRS was generated based on 12 risk variants by summing the number of risk alleles (zero, one, or two) for variants and subjects were stratified into five groups considering the distribution of GRS [41]. Correlation of GRS with GDM susceptibility was tested and we found that the GRS > 4 group had a nearly 1.80-fold increased susceptibility to GDM (adjusted OR 1.80 [95% CI 1.24–2.63]) than that of the lowest GRS group. After analysis of restricted cubic spline (RCS) regression between GRS and metabolic traits, only total cholesterol showed a significant nonlinear relationship between GRS (*P* for nonlinearity = 0.0038) [Suppl. Figure 3F]. Association between GRS groups and metabolic traits was evaluated and individuals with higher GRS had higher FPG and G_AUC_ and poorer β-cell function. We further used the effect sizes from the single SNP analysis (Table 2) to construct a weighted GRS. Restricted cubic spline (RCS) analysis showed a significant nonlinear relationship between weighted GRS and GDM (*P* for nonlinearity = 0.0133) [Suppl. Figure 4], according to which 0 and 10 were selected as the cutoff values of weighted GRS. Subjects with weighted GRS > 10 had a nearly 2.22-fold increased susceptibility to GDM (adjusted OR 2.22 [95% CI 1.64–2.99]) than GRS < 0 group [Suppl. Figure 5]. Our results highlight the value of the GRS based on the EETs pathway in GDM risk assessment in the Chinese population.

Complementing simple additive main effects of individual loci, gene-gene interactions (epistasis) can explain some of the unexplained heritability of common diseases [57]. Fisher [58] defined epistasis in a statistical manner as an explanation for deviation from additivity in a linear model. In our study, a significant interaction (epistasis) was identified between *CYP2J2*-rs76271683 and *CYP2C8*-rs11572177, suggesting that individuals carrying variants of both genes might be more susceptible to GDM than those carrying variants in single genes. However, statistical interaction does not necessarily imply interaction on the biological or mechanistic level and it is a challenge to go from a population-level statistical gene-gene interaction to the biological interactions occurring at the cellular level [59]. But they do suggest directions for the discovery of biological interactions.

To the best of our knowledge, this is the first study to suggest a genetic association between polymorphisms in the EET pathway and GDM, which can provide novel insights into the genetic architecture and aetiology of GDM. This study has several limitations. First, it was conducted only in Chinese population, which could lead to inherent bias or ethnicity-specific observations. Thus, further studies in other ethnic populations are needed. Second, while the analysis of genetic polymorphism in EET metabolic pathways supports its association with GDM and indicates possible risk factors, the potential relationship between enzyme activity and EET levels and its roles in the pathogenesis of GDM remain to be investigated. Therefore, further functional assays are necessary to explore the underlying functions and mechanisms of these polymorphisms. Third, lifestyle factors, such as cigarette smoking and dietary pattern were not included in the genotype-disease analysis. Prudent dietary pattern which is characterised by a high intake of fruit, green leafy vegetables, poultry, and fish was significantly and inversely associated with GDM risk and smoking has been found to increase the risk of developing GDM [60–62]. However, whether interactions existed between lifestyle factors and genetic variants remains unknown. Finally, we adopted an unusual threshold (r^2^ < 0.49) for SNPs selection, which may have caused the inclusion of non-independent SNPs in the GRS model and may have inflated the estimates.

## Conclusion

Our study suggests that common SNPs in key genes of EET processing and degradation pathways are associated with GDM in Chinese population. The GRS based on 12 independent SNPs was found to be positively associated with GDM. The mechanisms by which *CYP2C8*, *CYP2C9*, *CYP2J2*, and *EPHX2* influence GDM susceptibility warrant further investigation.

## Electronic supplementary material

Below is the link to the electronic supplementary material.

Supplementary Material 1

## Data Availability

The datasets used or analyzed during the current study are available from the corresponding author (Cheng Hu) on reasonable request.
