# Supplementary material for: Genetic variants in epoxyeicosatrienoic acid processing and degradation pathways are associated with gestational diabetes mellitus
Source: Nutr J. 2023 Jun 28;22:31. doi: 10.1186/s12937-023-00862-9 (PMC10303330; doi:10.1186/s12937-023-00862-9)
Supplement: Supplementary file 1 — Supplementary Material 1 [file 12937_2023_862_MOESM1_ESM.docx]

**Supplementary Information**


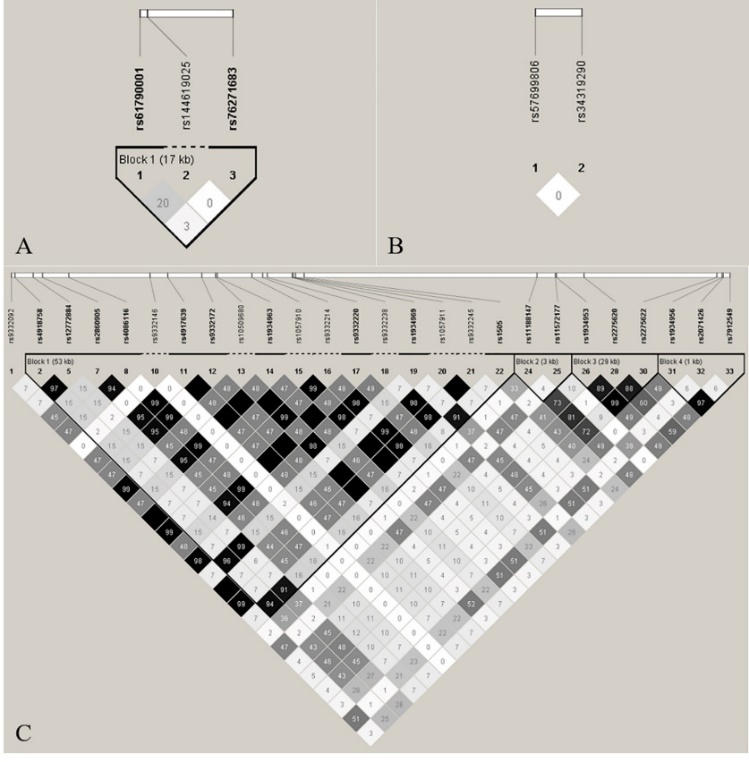


Suppl.Figure 1 Linkage disequilibrium pattern of *CYP2J2* (A), *EPHX2* (B), *CYP2C8*, and *CYP2C9* (C). Thirty-one SNPs were included in the LD analysis using the statistics r^2^ values. The colour of the squares ranges from light to dark, representing the degree of linkage from low to high. The numbers inside the squares refer to r^2^ value multiplied by 100.

Suppl. Table 1 Minor allele frequency of 12 SNPs related to key genes involved in EET processing and degradation pathways in different populations.

|  |  |  | African |  | American |  | European |  | South Asian |  | East Asian |  | Our study |
| --- | --- | --- | --- | --- | --- | --- | --- | --- | --- | --- | --- | --- | --- |
| *GENE* | SNP | Chr:bp | Minor allele/freq |  | Minor allele/freq |  | Minor allele/freq |  | Minor allele/freq |  | Minor allele/freq |  | Minor allele/freq |
| *CYP2J2* | rs61790001 | 1:59936155 | A: 0.016 |  | A: 0.069 |  | A: 0.099 |  | A: 0.211 |  | A: 0.131 |  | A: 0.153 |
|  | rs144619025 | 1:59937579 | T: 0 |  | T: 0 |  | T: 0 |  | T: 0 |  | T: 0.020 |  | T: 0.035 |
|  | rs76271683 | 1:59953494 | G: 0 |  | G: 0 |  | G: 0 |  | G: 0.001 |  | G: 0.173 |  | G: 0.167 |
| *EPHX2* | rs34319290 | 8:27405207 | T: 0.005 |  | T: 0.050 |  | T: 0.063 |  | T: 0.054 |  | T: 0.098 |  | T: 0.098 |
|  | rs57699806 | 8:27362587 | A: 0 |  | A: 0 |  | A: 0 |  | A: 0.003 |  | A: 0.061 |  | A: 0.047 |
| *CYP2C8* | rs11572177 | 10:96797270 | C: 0.333 |  | C: 0.199 |  | C: 0.331 |  | C: 0.259 |  | C: 0.073 |  | C: 0.076 |
|  | rs1934956 | 10:96828160 | T: 0.228 |  | T: 0.275 |  | T: 0.142 |  | T: 0.180 |  | T: 0.476 |  | T: 0.455 |
|  | rs2071426 | 10:96828323 | C: 0.256 |  | C: 0.151 |  | C: 0.283 |  | C: 0.220 |  | C: 0.059 |  | C: 0.068 |
| *CYP2C9* | rs2860905 | 10:96702295 | A: 0.272 |  | A: 0.170 |  | A: 0.236 |  | A: 0.189 |  | A: 0.093 |  | A: 0.096 |
|  | rs4918758 | 10:96697252 | C: 0.303 |  | C: 0.255 |  | C: 0.365 |  | C: 0.498 |  | C: 0.395 |  | C: 0.390 |
|  | rs9332092 | 10:96696529 | C: 0.002 |  | C: 0.037 |  | C: 0.073 |  | C: 0.110 |  | C: 0.033 |  | C: 0.045 |
|  | rs9332146 | 10:96722244 | A: 0.001 |  | A: 0.031 |  | A: 0.001 |  | A: 0 |  | A: 0.006 |  | A: 0.031 |


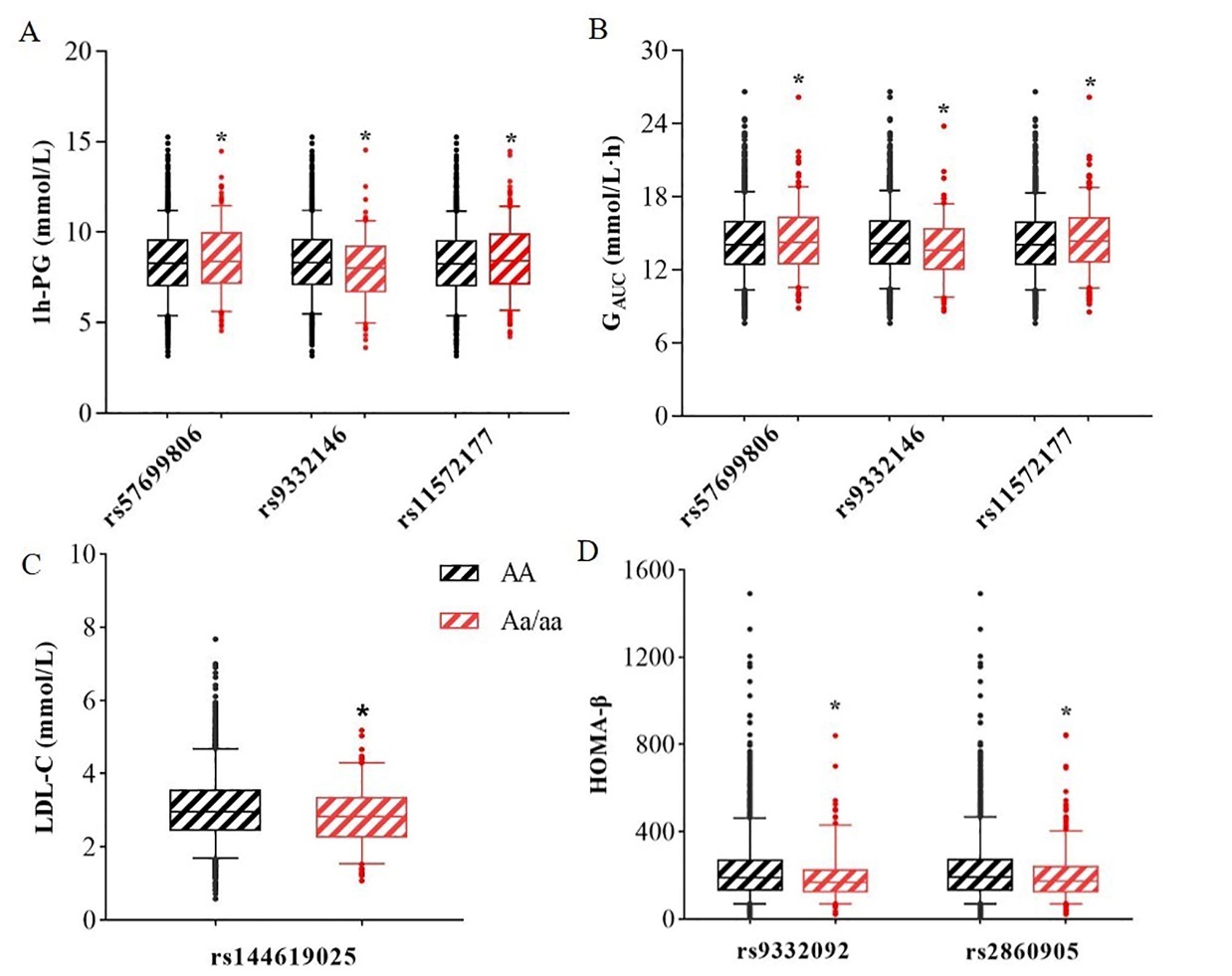


Suppl.Figure 2 Association between SNPs and metabolic traits. Box plot of 1h-PG (A), G_AUC_ (B), LDL-C (C) and HOMA-β (D) in different genotype groups in the whole study. A: major allele; a: minor allele.

Within each box, horizontal lines denote median values; boxes extend from the 25^th^ to the 75^th^ percentile of each group’s distribution of values; whiskers above and below the box indicate the 5^th^ and 95^th^ percentiles. Points above and below the whiskers indicate outliers.

**P* < 0.05 vs. AA group for each SNP after adjusted for maternal age, pre-pregnancy BMI, history of PCOS, history of GDM, and family history of diabetes, respectively.

Suppl. Table 2 Association of SNPs in EETs processing and degradation pathways with glycaemic traits (1)

|  |  | **FPG**（mmol/L） | | | | **1h-PG**（mmol/L） | | | | **2h-PG**（mmol/L） | | | | **G_AUC_**（mmol/L） | | | |
| --- | --- | --- | --- | --- | --- | --- | --- | --- | --- | --- | --- | --- | --- | --- | --- | --- | --- |
| **SNP** | ***Gene*** | **β** | **SE** | ***P*** | **FDR** | **β** | **SE** | ***P*** | **FDR** | **β** | **SE** | ***P*** | **FDR** | **β** | **SE** | ***P*** | **FDR** |
| rs61790001 | *CYP2J2* | 0.001 | 0.001 | 0.4379 | 0.6569 | -0.007 | 0.004 | 0.0564 | 0.0966 | -0.005 | 0.003 | 0.1959 | 0.2996 | -0.005 | 0.003 | 0.0804 | 0.1378 |
| rs144619025 | *CYP2J2* | 0.002 | 0.003 | 0.5078 | 0.6770 | -0.007 | 0.007 | 0.2857 | 0.3428 | -0.009 | 0.007 | 0.1890 | 0.2996 | -0.006 | 0.005 | 0.2542 | 0.3050 |
| rs76271683 | *CYP2J2* | 0.002 | 0.001 | 0.2270 | 0.011 | 0.012 | 0.003 | 0.0010 | **0.0125** | 0.011 | 0.003 | 0.0012 | **0.0145** | 0.010 | 0.003 | 0.0004 | **0.0047** |
| rs57699806 | *EPHX2* | 0.004 | 0.002 | 0.0952 | 0.5290 | 0.018 | 0.006 | 0.0040 | **0.0239** | 0.007 | 0.006 | 0.2497 | 0.2996 | 0.013 | 0.005 | 0.0075 | **0.0228** |
| rs34319290 | *EPHX2* | 0.003 | 0.002 | 0.1323 | 0.5290 | -2.18E-04 | 0.004 | 0.9604 | 0.9604 | 0.003 | 0.004 | 0.4433 | 0.4836 | 0.001 | 0.003 | 0.7241 | 0.7241 |
| rs9332092 | *CYP2C9* | 0.002 | 0.003 | 0.3809 | 0.6569 | 0.014 | 0.006 | 0.0314 | 0.0629 | 0.011 | 0.006 | 0.0810 | 0.1945 | 0.011 | 0.005 | 0.0239 | 0.0573 |
| rs4918758 | *CYP2C9* | 0.002 | 0.001 | 0.1112 | 0.5290 | 0.002 | 0.003 | 0.3530 | 0.3851 | 0.001 | 0.003 | 0.6531 | 0.6531 | 0.002 | 0.002 | 0.3174 | 0.3462 |
| rs2860905 | *CYP2C9* | 0.002 | 0.002 | 0.2208 | 0.5449 | 0.006 | 0.004 | 0.1712 | 0.2372 | 0.008 | 0.004 | 0.0591 | 0.1774 | 0.006 | 0.003 | 0.0942 | 0.1412 |
| rs9332146 | *CYP2C9* | -0.001 | 0.003 | 0.8459 | 0.8459 | -0.022 | 0.007 | 0.0067 | **0.0269** | -0.015 | 0.007 | 0.0359 | 0.1697 | -0.016 | 0.006 | 0.0076 | **0.0228** |
| rs11572177 | *CYP2C8* | 0.002 | 0.002 | 0.3895 | 0.6569 | 0.013 | 0.005 | 0.0098 | **0.0295** | 0.010 | 0.005 | 0.0424 | 0.1697 | 0.010 | 0.004 | 0.0075 | **0.0228** |
| rs1934956 | *CYP2C8* | -0.001 | 0.001 | 0.6539 | 0.7358 | -0.004 | 0.003 | 0.1779 | 0.2372 | -0.003 | 0.003 | 0.2365 | 0.2996 | -0.003 | 0.002 | 0.1515 | 0.2020 |
| rs2071426 | *CYP2C8* | 0.001 | 0.002 | 0.6745 | 0.7358 | 0.012 | 0.005 | 0.0226 | 0.0542 | 0.006 | 0.005 | 0.2021 | 0.2996 | 0.009 | 0.004 | 0.0298 | 0.0596 |

**Note:** All quantitative traits were log_10_-transformed before analysis. β, SE, and *P* values were obtained from linear regression analysis adjusted for maternal age, pre-pregnancy BMI, history of PCOS, history of GDM and family history of diabetes in the additive genetic model; FDR < 0.05 are written in bold letters.

**Abbreviations:** FPG, fasting plasma glucose; 1h-PG, oral glucose tolerance test (OGTT) one-hour plasma glucose; 2h-PG, OGTT two-hour plasma glucose; G_AUC_, area under the curve of glucose from the 75-g OGTT.

Suppl. Table 3 Association of SNPs in EETs processing and degradation pathways with glycaemic traits (2)

|  |  | **HbA1c** | | | | **Fasting insulin**（mU/L） | | | | **HOMA-β** | | | | **HOMA-IR** | | | |
| --- | --- | --- | --- | --- | --- | --- | --- | --- | --- | --- | --- | --- | --- | --- | --- | --- | --- |
| **SNP** | ***Gene*** | **β** | **SE** | ***P*** | **FDR** | **β** | **SE** | ***P*** | **FDR** | **β** | **SE** | ***P*** | **FDR** | **β** | **SE** | ***P*** | **FDR** |
| rs61790001 | *CYP2J2* | -0.002 | 0.001 | *0.1436* | 0.4204 | -0.003 | 0.009 | *0.7074* | 0.8280 | -0.010 | 0.010 | *0.3030* | 0.6746 | -0.002 | 0.009 | *0.8371* | 0.9132 |
| rs144619025 | *CYP2J2* | -0.001 | 0.002 | *0.5780* | 0.7706 | 0.008 | 0.018 | *0.6531* | 0.8280 | -0.001 | 0.019 | *0.9769* | 0.9769 | 0.011 | 0.019 | *0.5642* | 0.7699 |
| rs76271683 | *CYP2J2* | -0.001 | 0.001 | *0.5117* | 0.7675 | -0.001 | 0.009 | *0.9286* | 0.9286 | -0.008 | 0.010 | *0.3859* | 0.6746 | 0.001 | 0.009 | *0.9232* | 0.9232 |
| rs57699806 | *EPHX2* | 0.005 | 0.002 | *0.0043* | 0.0518 | 0.008 | 0.016 | *0.6125* | 0.8280 | -0.005 | 0.017 | *0.7682* | 0.8800 | 0.012 | 0.016 | *0.4600* | 0.7699 |
| rs34319290 | *EPHX2* | 1.32E-04 | 0.001 | *0.9176* | 0.9666 | 0.013 | 0.011 | *0.2483* | 0.8280 | 0.003 | 0.012 | *0.8067* | 0.8800 | 0.015 | 0.012 | *0.2045* | 0.7699 |
| rs9332092 | *CYP2C9* | -0.003 | 0.002 | *0.1778* | 0.4204 | -0.038 | 0.016 | *0.0190* | 0.1314 | -0.048 | 0.018 | *0.0076* | **0.0455** | -0.036 | 0.017 | *0.0341* | 0.2578 |
| rs4918758 | *CYP2C9* | 0.001 | 0.001 | *0.1615* | 0.4204 | 0.002 | 0.007 | *0.7590* | 0.8280 | -0.005 | 0.007 | *0.4747* | 0.6746 | 0.004 | 0.007 | *0.5759* | 0.7699 |
| rs2860905 | *CYP2C9* | 3.45E-04 | 0.001 | *0.7892* | 0.9470 | -0.026 | 0.011 | *0.0219* | 0.1314 | -0.034 | 0.012 | *0.0056* | **0.0455** | -0.024 | 0.012 | *0.0430* | 0.2578 |
| rs9332146 | *CYP2C9* | -0.002 | 0.002 | *0.4488* | 0.7675 | 0.011 | 0.019 | *0.5467* | 0.8280 | 0.014 | 0.021 | *0.5059* | 0.6746 | 0.011 | 0.020 | *0.5775* | 0.7699 |
| rs11572177 | *CYP2C8* | -0.002 | 0.001 | *0.1874* | 0.4204 | -0.011 | 0.012 | *0.3711* | 0.8280 | -0.019 | 0.014 | *0.1589* | 0.6357 | -0.009 | 0.013 | *0.4815* | 0.7699 |
| rs1934956 | *CYP2C8* | -3.22E-05 | 0.001 | *0.9666* | 0.9666 | 0.002 | 0.007 | *0.7323* | 0.8280 | 0.005 | 0.007 | *0.4706* | 0.6746 | 0.002 | 0.007 | *0.7990* | 0.9132 |
| rs2071426 | *CYP2C8* | -0.002 | 0.002 | *0.2102* | 0.4204 | -0.009 | 0.013 | *0.4985* | 0.8280 | -0.015 | 0.014 | *0.3054* | 0.6746 | -0.008 | 0.014 | *0.5622* | 0.7699 |

**Abbreviations:** HbA1c, haemoglobin A1c; HOMA-β, homeostasis model assessment index of β-cell secretion; HOMA-IR, homeostasis model assessment of insulin resistance.

FDR < 0.05 are written in bold letters.

Suppl. Table 4 Association of SNPs in EETs processing and degradation pathways with blood lipid metabolism-related traits (3)

|  |  | **Total cholesterol**（mmol/L） | | | | **Triglyceride**（mmol/L） | | | | **LDL-C**（mmol/L） | | | | **HDL-C**（mmol/L） | | | |
| --- | --- | --- | --- | --- | --- | --- | --- | --- | --- | --- | --- | --- | --- | --- | --- | --- | --- |
| **SNP** | **Gene** | **β** | **SE** | ***P*** | **FDR** | **β** | **SE** | ***P*** | **FDR** | **β** | **SE** | ***P*** | **FDR** | **β** | **SE** | ***P*** | **FDR** |
| rs61790001 | *CYP2J2* | -0.002 | 0.004 | *0.7201* | 0.8282 | 0.003 | 0.006 | *0.6272* | 0.8363 | -0.006 | 0.005 | *0.2800* | 0.5967 | -0.004 | 0.004 | *0.3971* | 0.7943 |
| rs144619025 | *CYP2J2* | -0.018 | 0.008 | *0.0301* | 0.1205 | 0.003 | 0.013 | *0.7872* | 0.9372 | -0.033 | 0.010 | *0.0014* | **0.0164** | -0.001 | 0.008 | *0.9236* | 0.9236 |
| rs76271683 | *CYP2J2* | 0.001 | 0.004 | *0.8282* | 0.8282 | 0.004 | 0.006 | *0.5508* | 0.8363 | 0.002 | 0.005 | *0.7647* | 0.7647 | 0.007 | 0.004 | *0.1186* | 0.7187 |
| rs57699806 | *EPHX2* | 0.009 | 0.007 | *0.2056* | 0.3524 | 0.012 | 0.011 | *0.2825* | 0.8363 | 0.004 | 0.009 | *0.6567* | 0.7647 | -0.011 | 0.007 | *0.1456* | 0.7187 |
| rs34319290 | *EPHX2* | 0.004 | 0.005 | *0.5013* | 0.6684 | 0.001 | 0.008 | *0.8680* | 0.9372 | 0.002 | 0.007 | *0.7231* | 0.7647 | 0.006 | 0.005 | *0.3038* | 0.7797 |
| rs9332092 | *CYP2C9* | -0.013 | 0.008 | *0.0968* | 0.2322 | 0.011 | 0.012 | *0.3548* | 0.8363 | -0.004 | 0.010 | *0.7054* | 0.7647 | 0.002 | 0.008 | *0.7783* | 0.9236 |
| rs4918758 | *CYP2C9* | -0.001 | 0.003 | *0.8256* | 0.8282 | 0.005 | 0.005 | *0.2718* | 0.8363 | -0.004 | 0.004 | *0.3247* | 0.5967 | 0.003 | 0.003 | *0.3249* | 0.7797 |
| rs2860905 | *CYP2C9* | -0.009 | 0.005 | *0.0790* | 0.2322 | 0.005 | 0.008 | *0.5064* | 0.8363 | -0.006 | 0.007 | *0.3450* | 0.5967 | -0.007 | 0.005 | *0.1797* | 0.7187 |
| rs9332146 | *CYP2C9* | -0.006 | 0.009 | *0.4734* | 0.6684 | -0.011 | 0.013 | *0.4184* | 0.8363 | -0.004 | 0.011 | *0.7431* | 0.7647 | -0.002 | 0.009 | *0.8631* | 0.9236 |
| rs11572177 | *CYP2C8* | -0.014 | 0.006 | *0.0172* | 0.1030 | 0.001 | 0.009 | *0.9372* | 0.9372 | -0.008 | 0.007 | *0.2760* | 0.5967 | -0.001 | 0.006 | *0.8707* | 0.9236 |
| rs1934956 | *CYP2C8* | 0.005 | 0.003 | *0.1307* | 0.2615 | 0.003 | 0.005 | *0.5735* | 0.8363 | 0.006 | 0.004 | *0.1155* | 0.5967 | -0.001 | 0.003 | *0.7307* | 0.9236 |
| rs2071426 | *CYP2C8* | -0.017 | 0.006 | *0.0073* | 0.0877 | -0.009 | 0.009 | *0.3541* | 0.8363 | -0.007 | 0.008 | *0.3481* | 0.5967 | -0.002 | 0.006 | *0.7175* | 0.9236 |

**Abbreviations:** LDL-C, low-density lipoprotein cholesterol; HDL-C, high-density lipoprotein cholesterol.

FDR < 0.05 are written in bold letters.

Suppl. Table 5 Interaction between key genes involved in EET processing and degradation pathways.

| CHR1 | SNP1 | CHR2 | SNP2 | OR_INT | STAT | *P* |  | CHR1 | SNP1 | CHR2 | SNP2 | OR_INT | STAT | *P* |  | CHR1 | SNP1 | CHR2 | SNP2 | OR_INT | STAT | *P* |
| --- | --- | --- | --- | --- | --- | --- | --- | --- | --- | --- | --- | --- | --- | --- | --- | --- | --- | --- | --- | --- | --- | --- |
| 1 | rs76271683 | 10 | rs11572177 | 0.61 | 6.10 | ***0.014*** |  | 1 | rs61790001 | 10 | rs1934956 | 0.87 | 1.29 | *0.256* |  | 8 | rs57699806 | 10 | rs1934956 | 0.99 | 0.00 | *0.975* |
| 1 | rs76271683 | 10 | rs9332092 | 0.62 | 3.17 | *0.075* |  | 1 | rs61790001 | 10 | rs9332092 | 0.78 | 0.68 | *0.410* |  | 10 | rs9332092 | 10 | rs2071426 | 0.56 | 1.89 | *0.169* |
| 1 | rs76271683 | 8 | rs34319290 | 0.76 | 2.07 | *0.150* |  | 1 | rs61790001 | 1 | rs76271683 | 1.18 | 0.66 | *0.417* |  | 10 | rs9332092 | 10 | rs1934956 | 1.14 | 0.24 | *0.623* |
| 1 | rs76271683 | 10 | rs2071426 | 0.77 | 1.63 | *0.201* |  | 1 | rs61790001 | 10 | rs9332146 | 1.32 | 0.57 | *0.449* |  | 10 | rs9332092 | 10 | rs4918758 | 0.88 | 0.18 | *0.674* |
| 1 | rs76271683 | 10 | rs2860905 | 0.81 | 1.32 | *0.251* |  | 1 | rs61790001 | 10 | rs11572177 | 0.88 | 0.36 | *0.551* |  | 10 | rs9332092 | 10 | rs11572177 | 0.84 | 0.17 | *0.679* |
| 1 | rs76271683 | 10 | rs9332146 | 0.69 | 1.15 | *0.284* |  | 1 | rs61790001 | 8 | rs57699806 | 1.15 | 0.27 | *0.606* |  | 10 | rs9332092 | 10 | rs2860905 | 1.13 | 0.04 | *0.846* |
| 1 | rs76271683 | 10 | rs4918758 | 0.91 | 0.66 | *0.416* |  | 1 | rs61790001 | 1 | rs144619025 | 1.25 | 0.26 | *0.607* |  | 10 | rs9332092 | 10 | rs9332146 | 0.97 | 0.00 | *0.969* |
| 1 | rs76271683 | 8 | rs57699806 | 0.92 | 0.11 | *0.744* |  | 1 | rs61790001 | 10 | rs2860905 | 1.10 | 0.25 | *0.618* |  | 10 | rs9332146 | 10 | rs1934956 | 1.16 | 0.30 | *0.586* |
| 1 | rs76271683 | 10 | rs1934956 | 1.01 | 0.01 | *0.907* |  | 8 | rs34319290 | 10 | rs1934956 | 1.30 | 3.59 | *0.058* |  | 10 | rs9332146 | 10 | rs2071426 | 1.16 | 0.06 | *0.808* |
| 1 | rs61790001 | 8 | rs34319290 | 0.94 | 0.10 | *0.751* |  | 8 | rs34319290 | 10 | rs11572177 | 0.95 | 0.05 | *0.829* |  | 10 | rs9332146 | 10 | rs11572177 | 0.94 | 0.01 | *0.927* |
| 1 | rs61790001 | 10 | rs4918758 | 1.02 | 0.04 | *0.838* |  | 8 | rs34319290 | 10 | rs4918758 | 0.78 | 2.95 | *0.086* |  | 10 | rs11572177 | 10 | rs1934956 | 1.13 | 0.61 | *0.436* |
| 1 | rs144619025 | 10 | rs1934956 | 0.72 | 1.89 | *0.169* |  | 8 | rs34319290 | 10 | rs9332146 | 0.58 | 1.29 | *0.257* |  | 10 | rs11572177 | 10 | rs2071426 | 0.94 | 0.07 | *0.793* |
| 1 | rs144619025 | 8 | rs57699806 | 1.82 | 1.56 | *0.212* |  | 8 | rs34319290 | 10 | rs2860905 | 0.82 | 0.65 | *0.419* |  | 10 | rs1934956 | 10 | rs2071426 | 1.33 | 1.71 | *0.192* |
| 1 | rs144619025 | 10 | rs11572177 | 0.53 | 1.26 | *0.262* |  | 8 | rs34319290 | 10 | rs9332092 | 0.86 | 0.19 | *0.664* |  | 10 | rs2860905 | 10 | rs2071426 | 0.68 | 2.91 | *0.088* |
| 1 | rs144619025 | 1 | rs76271683 | 1.26 | 0.34 | *0.562* |  | 8 | rs34319290 | 10 | rs2071426 | 1.11 | 0.15 | *0.697* |  | 10 | rs2860905 | 10 | rs11572177 | 0.81 | 1.01 | *0.316* |
| 1 | rs144619025 | 10 | rs2071426 | 0.84 | 0.11 | *0.740* |  | 8 | rs57699806 | 10 | rs9332092 | 1.88 | 1.96 | *0.162* |  | 10 | rs2860905 | 10 | rs9332146 | 1.34 | 0.23 | *0.634* |
| 1 | rs144619025 | 10 | rs9332146 | 0.77 | 0.10 | *0.751* |  | 8 | rs57699806 | 10 | rs2860905 | 1.52 | 1.81 | *0.178* |  | 10 | rs2860905 | 10 | rs1934956 | 0.98 | 0.01 | *0.928* |
| 1 | rs144619025 | 8 | rs34319290 | 0.87 | 0.09 | *0.765* |  | 8 | rs57699806 | 10 | rs4918758 | 1.23 | 1.07 | *0.302* |  | 10 | rs4918758 | 10 | rs11572177 | 0.77 | 2.54 | *0.111* |
| 1 | rs144619025 | 10 | rs4918758 | 0.94 | 0.07 | *0.788* |  | 8 | rs57699806 | 10 | rs11572177 | 1.23 | 0.31 | *0.578* |  | 10 | rs4918758 | 10 | rs1934956 | 0.94 | 0.56 | *0.455* |
| 1 | rs144619025 | 10 | rs2860905 | 1.06 | 0.02 | *0.884* |  | 8 | rs57699806 | 10 | rs9332146 | 0.65 | 0.25 | *0.615* |  | 10 | rs4918758 | 10 | rs9332146 | 1.29 | 0.46 | *0.497* |
| 1 | rs144619025 | 10 | rs9332092 | 1.00 | 0.00 | *0.998* |  | 8 | rs57699806 | 10 | rs2071426 | 0.88 | 0.12 | *0.731* |  | 10 | rs4918758 | 10 | rs2860905 | 1.02 | 0.02 | *0.895* |
| 1 | rs61790001 | 10 | rs2071426 | 0.74 | 1.65 | *0.199* |  | 8 | rs57699806 | 8 | rs34319290 | 0.96 | 0.01 | *0.910* |  | 10 | rs4918758 | 10 | rs2071426 | 1.00 | 0.00 | *0.980* |

Note: OR_INT refers to odds ratio of interaction. *P* < 0.05 are show in bold.


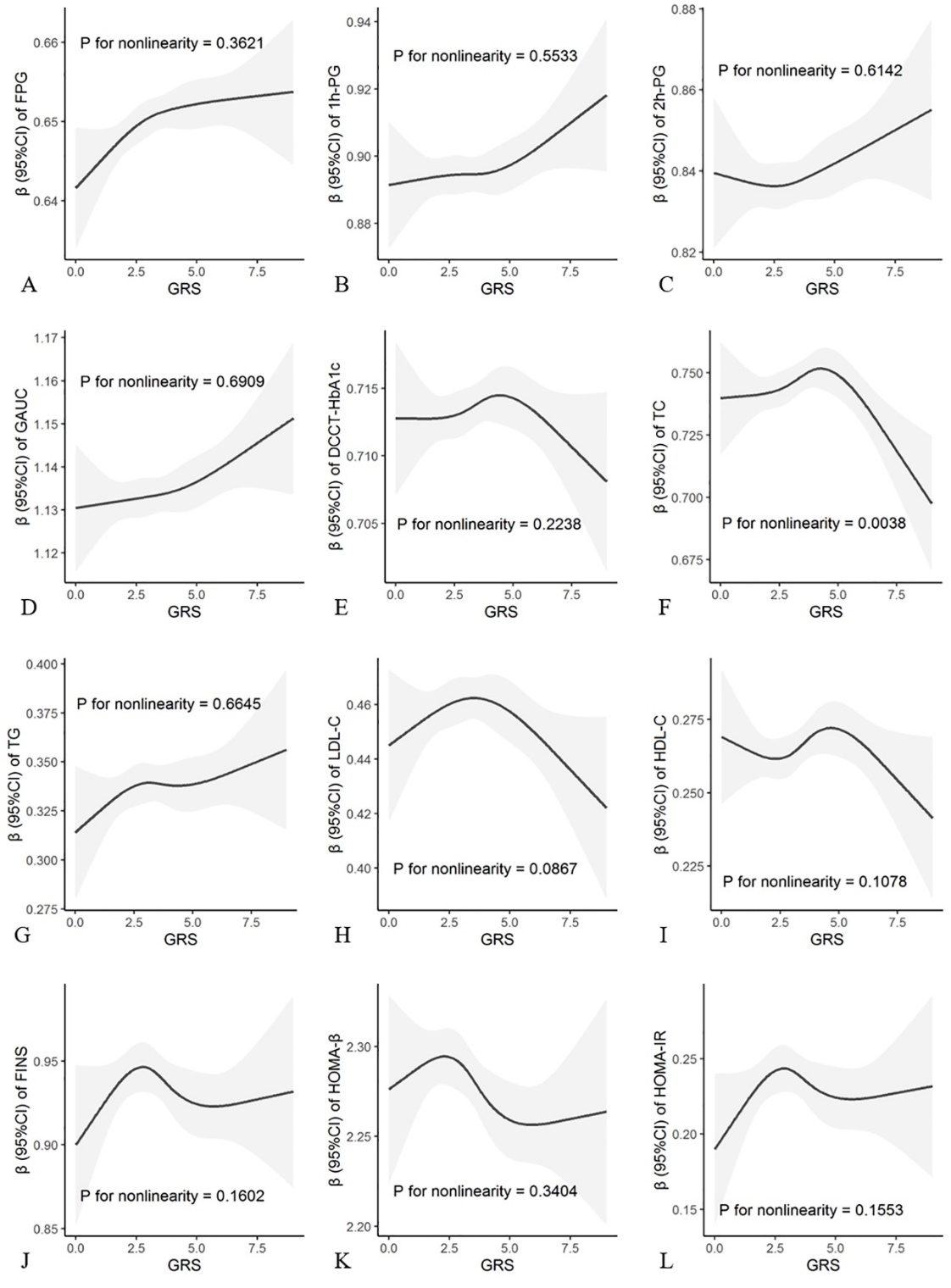


Suppl.Figure 3 Analysis of Restricted Cubic Spline Regression between simple GRS and metabolic traits.Model adjusted for maternal age, pre-pregnancy BMI, history of PCOS, history of GDM, and family history of diabetes. The solid lines indicate the estimated regression coefficient beta and the shaded region represents its 95% confidence interval.

**
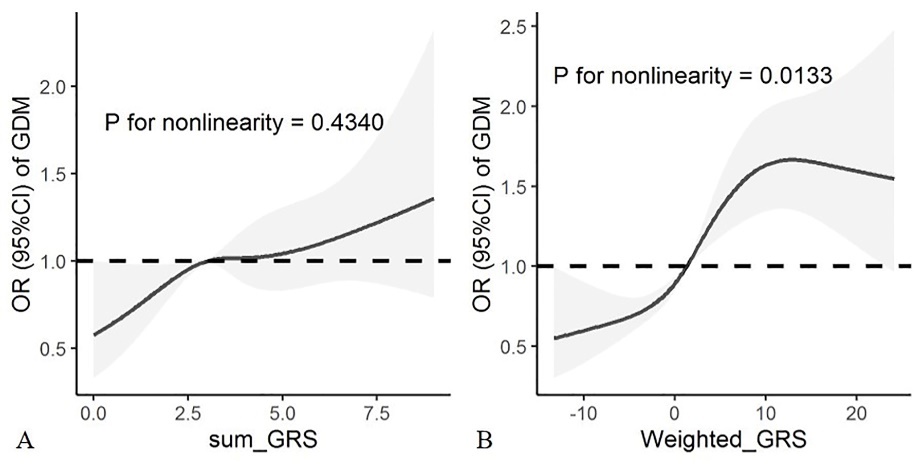
**

Suppl.Figure 4 Analysis of Restricted Cubic Spline Regression between GRS and GDM. Model adjusted for maternal age, pre-pregnancy BMI, history of PCOS, history of GDM, and family history of diabetes. The solid line indicates the adjusted odds ratio and the shaded region represents its 95% confidence interval. (A) sum_GRS: simple count GRS. (B) Weighted GRS.

**
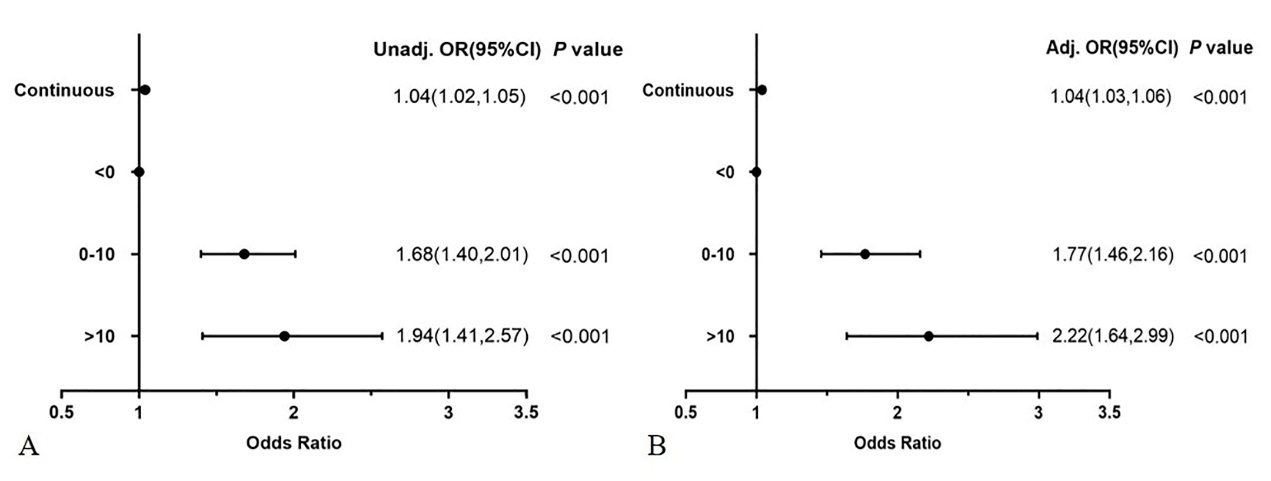
**

Suppl.Figure 5 Forest plot showing multivariable logistic regression analysis of weighted GRS and GDM. Covariates include maternal age, pre-pregnancy BMI, history of PCOS, history of GDM, and family history of diabetes.
